# Effect of a micronutrient‐rich snack taken preconceptionally and throughout pregnancy on ultrasound measures of fetal growth: The Mumbai Maternal Nutrition Project (MMNP)

**DOI:** 10.1111/mcn.12441

**Published:** 2017-03-02

**Authors:** Ashwin Lawande, Chiara Di Gravio, Ramesh D. Potdar, Sirazul A. Sahariah, Meera Gandhi, Harsha Chopra, Harshad Sane, Sarah H. Kehoe, Ella Marley‐Zagar, Barrie M. Margetts, Alan A. Jackson, Caroline H. D. Fall

**Affiliations:** ^1^ Dr Joshi Imaging Clinic Mumbai India; ^2^ MRC Lifecourse Epidemiology Unit University of Southampton Southampton UK; ^3^ Centre for the Study of Social Change Mumbai India; ^4^ Public Health Nutrition University of Southampton Southampton UK; ^5^ NIHR Southampton Biomedical Research Centre Southampton UK

**Keywords:** fetal growth, food‐based supplement, India, pregnancy, randomised controlled trial, ultrasound

## Abstract

Improving micronutrient intakes of under‐nourished mothers in low‐ and middle‐income countries increases birth weight, but there is little data on the nature and timing during gestation of any effects on fetal growth. Ultrasound measures of fetal size were used to determine whether and when a food‐based supplement affected fetal growth. Non‐pregnant women living in Mumbai slums, India (*N* = 6,513), were randomly assigned to receive either a daily micronutrient‐rich snack containing green leafy vegetables, fruit, and milk (treatment) or a snack made from lower‐micronutrient vegetables (control) in addition to their usual diet from before pregnancy until delivery. From 2,291 pregnancies, the analysis sample comprised 1,677 fetuses (1,335 fetuses of women supplemented for ≥3 months before conception). First‐trimester (median: 10 weeks, interquartile range: 9–12 weeks) fetal crown‐rump length was measured. Fetal head circumference, biparietal diameter, femur length, and abdominal circumference were measured during the second (19, 19–20 weeks) and third trimesters (29, 28–30 weeks). The intervention had no effect on fetal size or growth at any stage of pregnancy. In the second trimester, there were interactions between parity and allocation group for biparietal diameter (*p* = .02) and femur length (*p* = .04) with both being smaller among fetuses of primiparous women and larger among those of multiparous women, in the treatment group compared with the controls. Overall, a micronutrient‐rich supplement did not increase standard ultrasound measures of fetal size and growth at any stage of pregnancy. Additional ultrasound measures of fetal soft tissues (fat and muscle) may be informative.

## 1 INTRODUCTION

Low birth weight (LBW) is an important public health problem in low‐ and middle‐income countries (UNICEF & WHO, 2004). LBW commonly results from intrauterine growth restriction and is associated with increased neonatal mortality and morbidity, slower postnatal growth, poorer cognitive development, and a higher risk of chronic non‐communicable diseases in later life (Barker, 1998; Victora et al., 2008).

Poor maternal nutritional status contributes to the prevalence of LBW (Osrin & De L Costello, 2000). Many studies have investigated the effect on birth outcomes of supplementing mothers with multiple micronutrients during pregnancy (Haider & Bhutta, 2015; Kawai, Spiegelman, Shankar, & Fawzi, 2011). Meta‐analyses suggest that this reduces the prevalence of LBW (Fall, Fisher, Osmond, & Margetts, 2009; Shah, Ohlsson, & Knowledge Synthesis Group on Determinants of Low Birth Weight and Preterm Births, 2009). An observational study in Pune, India, showed positive associations between birth weight and the frequency of consumption by the mother of milk in early gestation, and of green leafy vegetables and fruit in late gestation, with the latter associations being stronger in lighter and thinner women (Rao et al., 2001).

The Pune results led to a randomised controlled trial, the Mumbai Maternal Nutrition Project (MMNP), to test whether supplementing the mother's diet with green leafy vegetables, milk, and fruit reduces LBW. Recent evidence indicates that processes occurring in early gestation, such as de‐ and re‐methylation of fetal DNA, the development of the placenta, and fetal organogenesis are important determinants of not only size at birth but also long‐term health (Cetin, Berti, & Calabrese, 2010; Oliver, Jaquiery, Bloomfield, & Harding, 2007; Watkins & Fleming, 2009). MMNP therefore aimed to enhance maternal nutritional status for a sustained period of time (chosen a priori to be at least 3 months) before conception as well as throughout pregnancy. Low‐income non‐pregnant women in Mumbai, who intended to have children, were randomly assigned to receive either a daily micronutrient‐rich snack containing green leafy vegetables, fruit, and milk or a lower‐micronutrient snack, in addition to their usual diet from before pregnancy until delivery (Potdar et al., 2014). There was no overall effect of the intervention on birth weight in the intention to treat analysis but a positive effect in the per protocol analysis limited to women supplemented for at least 3 months before conception. In both analyses, there was an interaction between allocation group and maternal pre‐pregnancy body mass index (BMI) such that, while there was no intervention effect in underweight women (BMI ≤18.5 kg/m^2^), there was a positive effect on birth weight among mothers of normal or high BMI.

Birth weight is not an optimal proxy for fetal growth, because two babies with the same birth weight and size may achieve this by different growth trajectories (Wills, Yajnik, & Kinare, 2010). We used ultrasound measures of fetal size in the MMNP to (a) determine whether supplementation influenced fetal size and growth and (b) determine the timing during pregnancy of any effect. We hypothesised that fetal measurements would be increased in the treatment group, that differences would be present from early pregnancy, and that effects would increase with maternal pre‐pregnancy BMI.

**Key messages**

- In this study, a food‐based micronutrient‐rich supplement had no overall effect on standard ultrasound measures of fetal size or growth.
- In a subgroup analysis the intervention may have increased growth in fetal biparietal diameter and femur length up to 20 weeks among multiparous mothers, and reduced it among primiparous mothers.
- To understand nutritional effects on fetal growth, additional ultrasound measures of fetal soft tissue (adipose tissue and muscle) might be informative.

## 2 METHODS

### 2.1 Study population

Enrolment into the MMNP took place between 2006 and 2012 in slum areas of Mumbai, India, covered by the health and social programs of the non‐governmental organization the Centre for the Study of Social Change. Women were eligible if aged <40 years, married, non‐pregnant, not sterilised, planning to have children and intending to deliver in Mumbai. Six thousand five hundred thirteen women were recruited and randomly assigned to receive either a daily micronutrient‐rich snack containing green leafy vegetables, fruit, and milk (treatment group) or a snack made from lower‐micronutrient vegetables such as onion and potato (controls), in addition to their usual diet, from before pregnancy until delivery. Random assignment was generated remotely in Southampton, United Kingdom. Women were individually randomly assigned, after stratifying by age and BMI.

To optimise the content and palatability of the supplements we carried out extensive pilot work before starting the trial (Shivashankaran et al., 2011). The most acceptable way of delivering the foods was in the form of a snack that resembled local street food (e.g., samosas and fritters). Treatment snacks contained fresh or dried green leafy vegetables, full‐fat milk powder, and fruit powder or dried fruit (Table S1). Multiple recipes were tested for palatability prior to the trial, and we continued to develop new recipes throughout the trial to reduce monotony and in response to the women's comments, with only small changes in micronutrient content; on average, treatment snacks contained 10%–23% of the WHO/FAO recommended Reference Nutrient Intake for β‐carotene, riboflavin, folate, vitamin B12, calcium, and iron compared with <10% in control snacks (Table S2). Further information on the randomization procedure and snacks can be found elsewhere (Potdar et al., 2014).

### 2.2 Data collection

Health workers made home visits to explain the trial, and community meetings were held to answer questions and obtain consent. Women were invited to attend recruitment clinics, at which they were screened for eligibility and individual written consent was obtained. At recruitment, women were asked about their occupation, education, religion, parity, and use of tobacco (in both smoked and chewed form). Socio‐economic status was assessed using the standard of living index, a widely used questionnaire‐based method developed for national surveys, based on housing type, utilities, and household possessions (International Institute for Population Sciences, 2001). A higher score represents higher socio‐economic status. Diet was assessed at recruitment and in the second trimester of pregnancy using a quantified food frequency questionnaire (Chopra et al., 2012) with a reference period of the preceding week. Weight and height were measured using standardised in‐house protocols. Height was measured to the nearest 0.1 cm using a portable Harpenden stadiometer (CMS Instruments Ltd. London) with the head positioned in the Frankfort plane. Weight was measured to the nearest 0.5 kg after removing heavy items of clothing and jewellery.

To ensure that women would not have to walk further than 300–500 m from home to obtain their snacks, 61 supplementation centres were set up in the study area. Women were given one snack a day, and consumption was observed and recorded. Women were deemed compliant if they consumed, on average, at least half the available snacks in a given week. Staff at the supplementation centres maintained a record of the women's last menstrual period (LMP) dates, and updated this every month.

Women who missed two periods had a urinary pregnancy test, and if this was positive, they were invited to a central clinic at Centre for the Study of Social Change at 9–12 weeks gestation for an obstetric assessment and ultrasonography to confirm the pregnancy and measure fetal size. Further ultrasound scans were scheduled for 19–21 and 28–32 weeks gestation. For the purpose of this study, in which we wanted to detect differences in fetal size, even in early stages of pregnancy, we based gestational age on LMP date rather than ultrasound measurements (Wills et al., 2010).

Fetal biometry was measured using a Siemens Sonoline ADARA ultrasound machine with a 4‐MHz probe. At visit 1, crown‐rump length (CRL) was measured. However, if women attended late and the gestational age at the first examination was ≥13 weeks, fetal head circumference (HC), biparietal diameter (BPD), femur length (FL), and abdominal circumference (AC) were recorded instead. HC, BPD, FL, and AC were assessed at the two subsequent visits. Measurements were performed using standard techniques (Hadlock, 1990). HC was calculated using the longest and shortest axes of the fetal head, measured from the outer to outer surfaces of the skull. BPD was measured from outer to inner surfaces of the skull. FL was measured along the long axis of the femur without the distal femoral epiphysis. AC was estimated using the anteroposterior and the transverse diameters (Hadlock, 1990); after ensuring that the stomach bubble was visible, the abdomen filled at least 30% of the monitor screen and neither the kidneys nor the bladder were visible, taking care not to cause distortion by exerting too much pressure with the probe (Papageorghiou et al., 2014). At each examination, HC, BPD, and FL were measured once. AC was measured once only if the fetus was optimally positioned to obtain a perfect view (80% of the total scans); in the remaining cases, AC was measured in triplicate and the average of the three measures was included in the analysis. Scans were carried out by a single operator (AL) throughout the trial.

Trained research nurses measured newborns within 10 days of birth. Measurements included weight (to the nearest 10 g, Seca scales) and occipito‐frontal head circumference and abdominal circumference immediately below the umbilicus, each measured thrice to the nearest 0.1 cm using fibreglass tapes and averaged.

### 2.3 Analysis sample

When we started the trial, pregnancies were followed up only if the women started supplementation at least 3 months prior to their LMP date. However, the exclusion of women who conceived within 3 months of starting supplementation disappointed the women concerned and threatened the community's goodwill towards the project, and so from December 2008, we followed up all pregnancies (Potdar et al., 2014). This change in protocol led to the implementation of two analyses: intention‐to‐treat (ITT) and per‐protocol (PP). The former included all pregnancies, whereas the latter was limited to women supplemented for 3 months or more before their LMP date.

**Figure 1:**
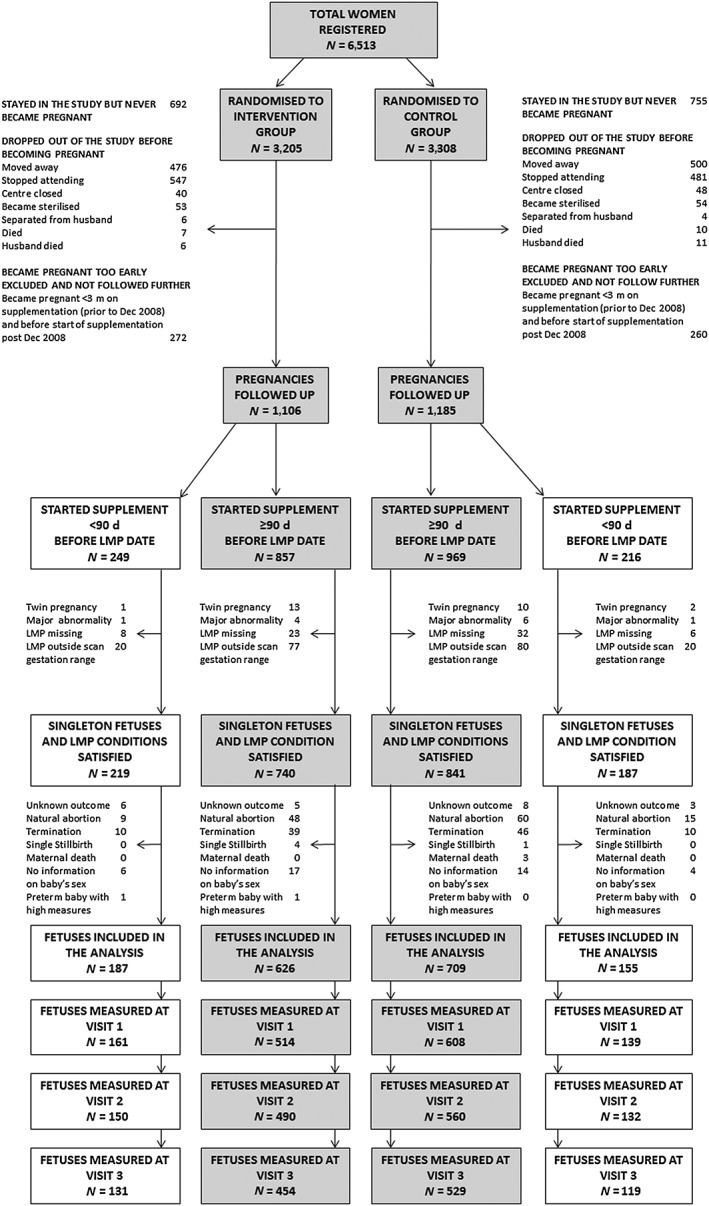
Flowchart of participants in the Mumbai Maternal Nutrition Project (MMNP). Shaded boxes indicate women who started supplementation ≥3 months before their last menstrual period (LMP)

For this analysis, we excluded twins (*n* = 26), fetuses with major congenital abnormalities (*n* = 12), and those with a missing maternal LMP date (*n* = 69). We also excluded pregnancies in which the LMP‐derived gestation differed by more than 2 weeks from the gestation estimated from an early (<20 weeks) ultrasound scan (*n* = 197), because the LMP date was likely to be inaccurate in these cases. Pregnant women with no information on delivery outcome (*n* = 22) and newborns with missing information on sex (*n* = 41) were also excluded; these were usually women who went to the village for delivery and were lost to follow‐up. In India, it is illegal to reveal the sex of the fetus during pregnancy, and so we had to exclude all pregnancies resulting in abortions, terminations, stillbirths, and maternal deaths (*n* = 245) because of unknown fetal sex. Two preterm babies (<37 weeks of gestation) whose gestational‐age‐adjusted fetal measures, at each scan, were >3 standard deviations (SDs) higher than the population mean were excluded because, given their available fetal biometric parameters, their LMP date was likely to be incorrect. The exclusion criteria reduced the initial sample to 1,677 pregnancies in the ITT analysis and 1,335 pregnancies in the PP analysis. Among those 90% (ITT analysis: 1,508; PP analysis: 1,197) had one or more ultrasound measures. The sample considered in this study is summarised in Figure 1.

### 2.4 ETHICS

The trial (ISRCTN62811278) was approved by the ethics committees of BYL Nair and TN Medical College, Grant Medical College, and Sir JJ Group of Hospitals, Mumbai, and Southampton and SW Local Research Ethics Committees. An independent data‐monitoring committee reviewed the data every 6 months for 2 years and then annually. The trial protocol can be obtained from the corresponding author.

### 2.5 STATISTICAL METHODS

We examined the differences in baseline measurements between women who had three scans and those who had two or fewer scans. We calculated partial correlations among gestation‐adjusted fetal size measures whilst controlling for sex and allocation group. We compared fetal biometry with the INTERGROWTH‐21^st^ standards (Papageorghiou et al., 2014) by computing the *z* score of HC, FL, and AC as

$$z\;{\text{score}}_{\text{MMNP}}=\frac{\text{MMNP observation}-\text{INTERGROWTH mean}}{\text{INTERGROWTH}\;\mathrm{SD}}$$

in the second (14–27 weeks) and the third trimester (>27 weeks) of pregnancy. We were not able to compare BPD in this way, because it was measured differently in the two studies.

To test the effect of the intervention on fetal size, we considered each visit separately and used all available data at each visit. Because gestational age at the time of each visit varied between women, and fetal size differed between the sexes (Table S3 and Figure S1), within cohort sex‐and‐gestation‐specific *z* scores were calculated using the Lambda‐Mu‐Sigma (LMS) method (Cole & Green, 1992; Fenton & Sauve, 2007). The effect of the intervention on fetal growth was analysed using mixed effects regression models to take into account the correlation between repeated observations in the same individual and the possibility of a nonlinear association between fetal biometry and gestational age.

Unadjusted comparisons of fetal measures between allocation groups were made using *t* tests and Mann–Whitney U tests for normally and non‐normally distributed variables, respectively. Multiple regression models were implemented to assess the effect of the intervention on fetal size and growth. The presence of interactions between allocation group and maternal pre‐pregnancy BMI, height and age (continuous variables), parity (discrete variable), and sex (binary variable) was evaluated. Interactions between allocation group and second trimester intakes of green leafy vegetables, fruit, and milk were considered when analysing the effect of the intervention on fetal size at visit 3. The effect of adjusting for compliance was also examined; for this purpose, average compliance was calculated from 3 months prior to the LMP (or from recruitment if supplementation <3 months) up to the visit of interest. Tobacco use was not included in the final set of adjustors because only 206 (9%) of pregnant women consumed tobacco (mostly in chewed form), and there were no associations between maternal tobacco use and fetal measurements. Women's occupation, education, and standard of living index score were first included as possible confounders; however, as there were no associations between those variables and fetal measures (results not shown), we excluded them from the models presented in this paper. Results were considered statistically significant when *p* < .05. The analyses were performed using R V.3.2.2 (Rigby & Stasinopoulos, 2005) and Stata V.14 (Stata Corporation, College Station, TX).

## 3 RESULTS

Two thousand two hundred ninety‐one women (35%) became pregnant during the trial and were followed up. The median age at conception was 25 years (interquartile range: 22–28); 34% of women were underweight (BMI < 18.5 kg/m^2^) while 9% were overweight (BMI between 25 and 29.9 kg/m^2^) and 2% were obese (BMI ≥ 30 kg/m^2^). The majority of women were not in paid work (79%), had completed secondary education (88%), were Hindu (70%), and spoke either Marathi or Gujarati as their first language (55%). Forty‐six percent of women were primiparous. Baseline characteristics of women who became pregnant are summarised in Table 1.

**Table 1:** Pre‐pregnant characteristics of the women who became pregnant and fetal measures, according to allocation group

	Treatment (*N* = 1,106)		Control (*N* = 1,185)		*p*
	Median (IQR) or *n* (%)	*N*	Median (IQR) or *n* (%)	*N*	
Weight (kg)	45.4 (39.8–51.6)	1,105	46 (40.6–52)	1,185	.17
Height (cm)^a^	151 (5.6)	1,106	151 (5.4)	1,184	.66
BMI (kg/m^2^)	19.8 (17.8–22.5)	1,105	19.9 (17.9–22.5)	1,184	.15
Age (years)^b^	25 (22–28)	1,105	25 (22–28)	1,180	.02
Parity		1,106		1,185	.04
0	384 (34.7%)		350 (29.5%)		
1	497 (44.9%)		564 (47.6%)		
2+	225 (20.3%)		271 (22.9%)		
Religion		1,106		1,184	.64
Hindu	785 (71.0%)		827 (69.9%)		
Muslim	285 (25.8%)		313 (26.4%)		
Other	36 (3.25%)		44 (3.70%)		
Education		1,105		1,184	.37
Primary	128 (11.6%)		116 (9.80%)		
Secondary	920 (83.3%)		1,003 (84.7%)		
Graduate	57 (5.16%)		65 (5.49%)		
Social living index score	25 (21–29)	1,077	25 (21–29)	1,138	.98
Mothertongue		1,105		1,182	
Marathi/Gujarati	614 (55.6%)		643 (54.4%)		
Hindi/Punjabi/Bengali	401 (36.3%)		450 (38.1%)		
Other	90 (8.14%)		89 (7.53%)		
Occupation		1,106		1,185	.61
Unskilled/semi‐skilled	171 (15.5%)		198 (16.7%)		
Skilled/self‐employed	29 (2.62%)		38 (3.21%)		
Semi‐professional/professional	20 (1.81%)		25 (2.11%)		
Not working	886 (80.1%)		924 (78.0%)		
Frequencies of dietary intake					
Milk and milk products (tea excluded)		1,106		1,185	.38
<1 time/week	557 (50.4%)		572 (48.3%)		
1–6 times/week	388 (35.1%)		449 (37.9%)		
≥7 times/week	161 (14.6%)		164 (13.8%)		
GLV		1,106		1,185	.60
<1 time/week	266 (24.1%)		278 (23.5%)		
1–6 times/week	880 (74.3%)		880 (74.3%)		
≥7 times/week	32 (2.89%)		27 (2.28%)		
Fruit		1,106		1,185	.48
<1 time/week	171 (15.5%)		204 (17.2%)		
1–6 times/week	755 (68.3%)		800 (67.5%)		
≥7 times/week	180 (16.3%)		181 (15.3%)		
Fetal Measures^c^^,^ d					
CRL (cm)	2.9 (2.5–3.3)	540	2.9 (2.5–3.3)	611	.67
HC (cm)					
Visit 1	9.6 (9.1–10.2)	135	9.8 (9.1–10.4)	132	.25
Visit 2	16.9 (16.0–17.8)	640	16.9 (16.0–17.9)	689	.44
Visit 3	27.9 (27.0–28.7)	581	27.9 (27.0–28.8)	643	.98
BPD (cm)					
Visit 1	2.6 (2.5–2.8)	135	2.6 (2.5–2.8)	136	.66
Visit 2	4.5 (4.4–4.9)	640	4.6 (4.4–4.9)	692	.31
Visit 3	7.6 (7.3–7.9)	585	7.6 (7.4–7.9)	648	.68
FL (cm)					
Visit 1	1.3 (1.1–1.4)	102	1.3 (1.2–1.5)	109	.13
Visit 2	3.2 (2.9–3.4)	638	3.1 (2.9–3.4)	690	.52
Visit 3	5.7 (5.5–5.9)	585	5.7 (5.4–5.9)	642	.17
AC (cm)					
Visit 1	7.6 (6.8–8.1)	110	7.6 (7.0–8.2)	114	.69
Visit 2	13.6 (12.8–14.6)	637	13.6 (12.8–14.5)	687	.43
Visit 3	23.8 (22.7–25.0)	583	23.9 (22.7–24.9)	645	.91
Birth measures^c^^,^ d					
Birth weight^a^ (g)	2,651 (375)	572	2,610 (394)	621	.25
HC^a^ (cm)	33.2 (1.3)	562	33.2 (1.3)	681	.61
AC^a^ (cm)	28.5 (2.2)	564	28.4 (2.1)	682	.54

*Note*. AC = abdominal circumference; BPD = biparietal diameter; CRL = crown‐rump length; FL = femur length; GLV = green leafy vegetables; HC = head circumference; IQR = interquartile range.

a Mean and standard deviation for normally distributed variable.

b Age at conception.

c Values adjusted for median gestational age (in weeks); includes only those pregnancies that satisfied the conditions imposed on the last menstrual period date.

d Singleton pregnancy without congenital abnormalities and with known sex and gestational age. Include only those pregnancies that satisfied the condition imposed on the last menstrual period date (LMP).

The median (interquartile range) gestational age at each examination was 10 (9–12), 19 (19–20) and 29 (28–30) weeks, respectively. Of the fetuses included in the analysis (*n* = 1,677), 1,151 (treatment: 67%, control: 71%) had CRL measured at visit 1, 1,332 fetuses (treatment: 74% control: 80%) were measured at visit 2 and 1,233 (treatment: 73%, control: 75%) were measured at visit 3. One thousand one hundred five (treatment: 47%, control: 49%) women had three scans recorded, 471 (treatment: 22%, control: 19%) had two, and 223 (treatment: 10%, control: 10%) had only a single scan. Baseline characteristics were mostly similar between women with complete data and those with one or more scan missing (Table S4); differences were observed with respect to parity and occupation. Specifically, women with one previous delivery had higher odds of having a full set of scans when compared to primiparous women (OR: 1.47, 95% CI [1.22, 1.78], *p* < .001), and nonworking women had lower odds than women engaged in paid work outside the home (OR: 0.71, 95% CI [0.58, 0.87], *p* = .001).

**Figure 2:**
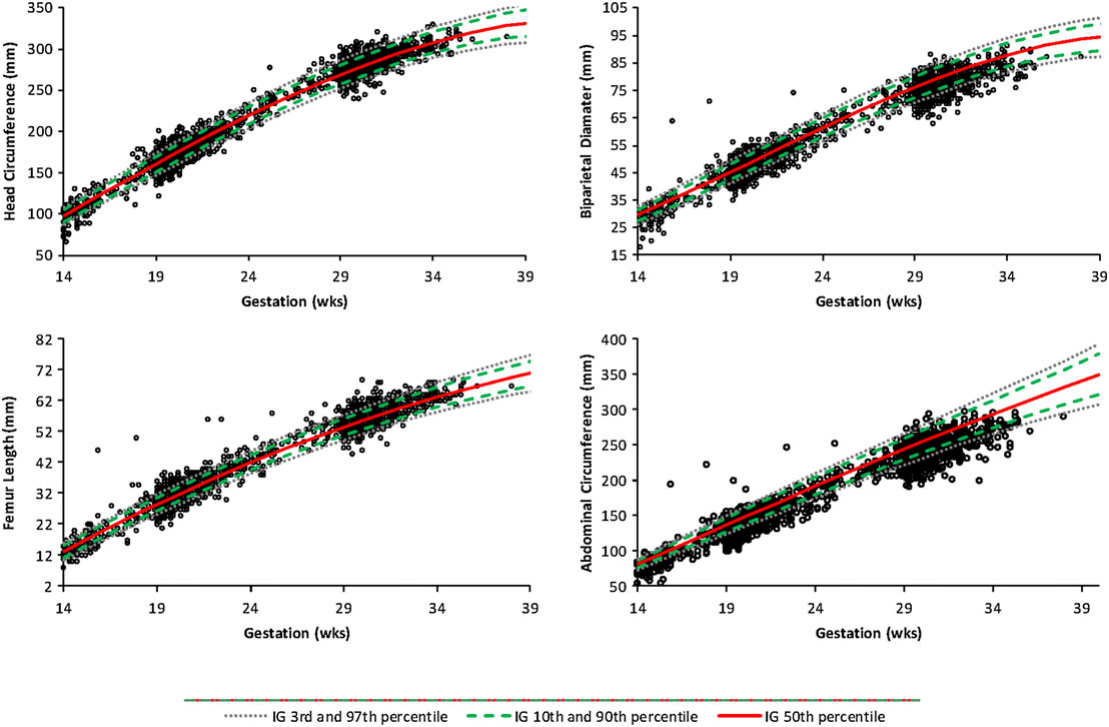
Head circumference, biparietal diameter, femur length, and abdominal circumference according to gestational age (weeks) in relation to the 3rd, 10th, 50th, 90th, and 97th centiles from the INTERGROWTH‐21^st^ standards. Biparietal diameter in our study was measured differently from the INTERGROWTH‐21^st^ project; hence, the value could not be formally compared with the provided international standards. IG = INTERGROWTH

Partial correlation coefficients among gestation‐adjusted fetal and newborn measures were positive and statistically significant (Table S5). Hence, fetuses who were larger in early gestation tended to be larger in the later stages of pregnancy and at birth. When compared to the INTERGROWTH‐21^st^ standards, fetal AC was significantly smaller in both the second and third trimesters of pregnancy (−1.21SD and −1.26SD, respectively), whereas HC (−0.03SD and 0.03SD, respectively) and FL (0.31SD and 0.36SD, respectively) were more comparable (Figure 2).

### 3.1 Intention‐to‐treat analysis

**Figure 3:**
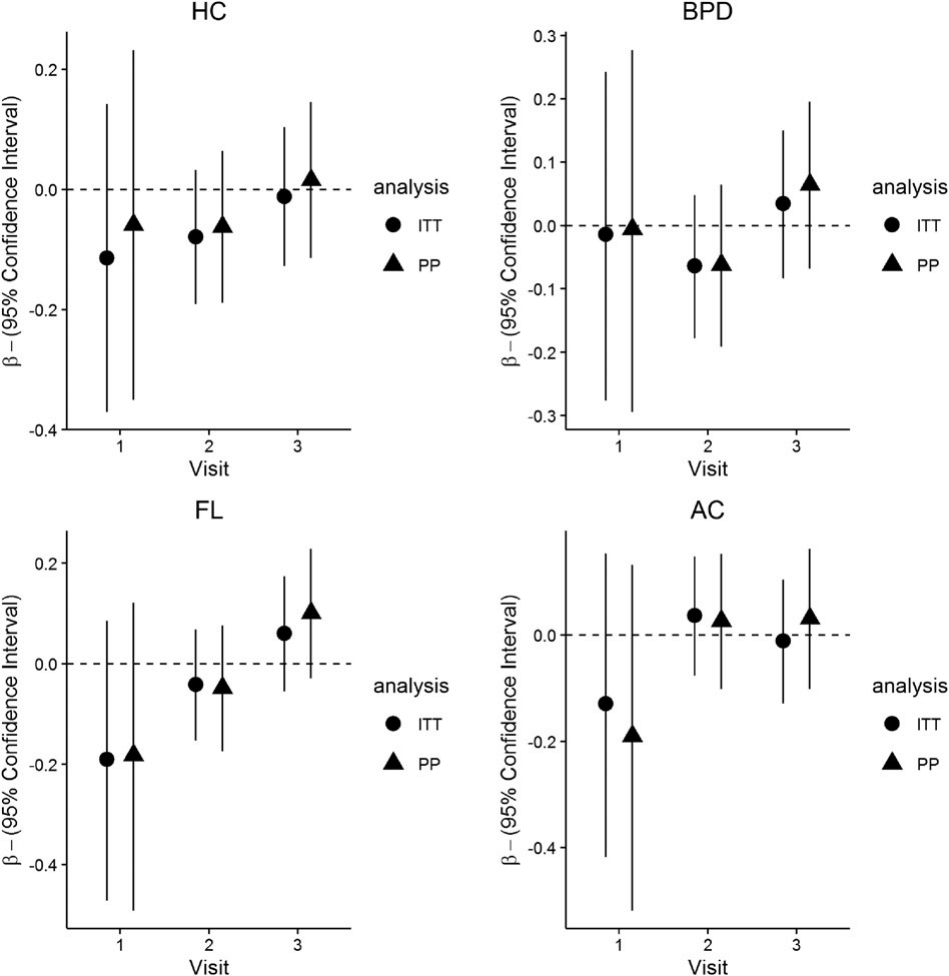
Standardised regression coefficients β (and 95% confidence interval) for the effect of the intervention on HC, BPD, FL, and AC at visits 1, 2, and 3. Results are taken from the unadjusted analysis (differences are computed as treatment − control. A positive β indicates larger size in the intervention group). Circles refer to the mean difference in fetal size between control and treatment groups in the intention‐to‐treat analysis. Triangles represents the same values estimated in the per‐protocol analysis. AC = abdominal circumference; BPD = biparietal diameter; FL = femur length; HC = head circumference; ITT = intention‐to‐treat; PP = per‐protocol

**Figure 4:**
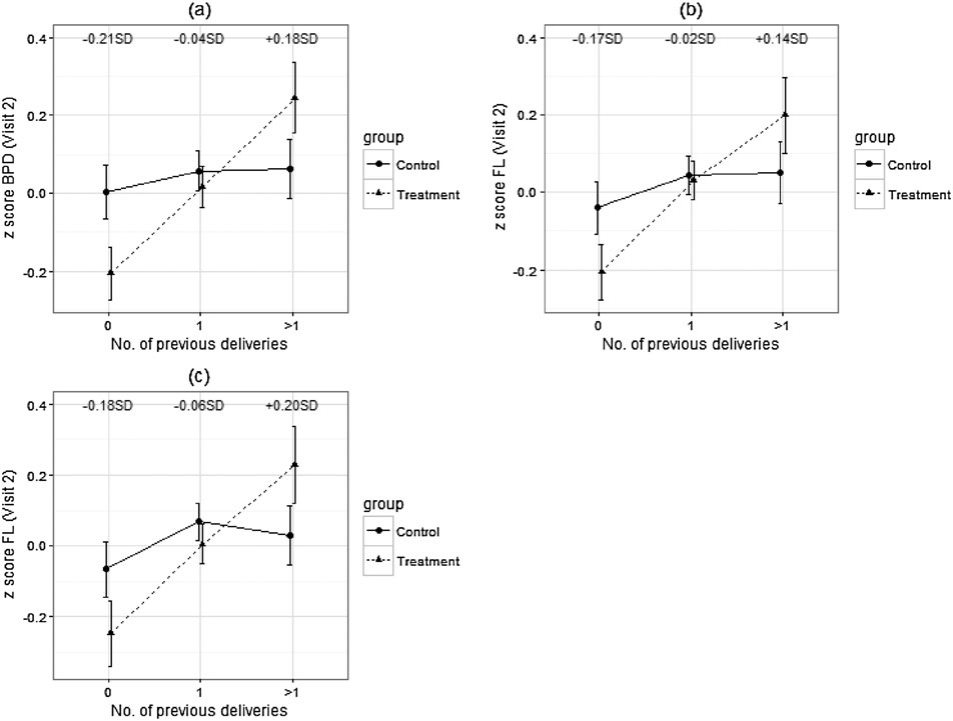
Effect of the intervention on BPD and FL at visit 2 according to maternal parity. Values are means; error bars indicate 95% confidence intervals. Panels (a) and (b) refer to the results obtained in the intention‐to‐treat analysis. Panel (c) summarises the results from the per‐protocol analysis (women who started supplementation ≥3 months before their last menstrual period date). The numbers in each figure summarise the mean difference between treatment and control of the considered measure according to parity. BPD = biparietal diameter; FL = femur length

In unadjusted analyses, there was no effect of the intervention on CRL at visit 1 (treatment mean CRL: −0.02SD; control mean CRL: 0.02SD; difference between means: 0.04SD, 95% CI [−0.08SD, 0.16SD]; *p* = .50) or on HC, BPD, FL, and AC at any of the visits separately (Figure 3). There were no significant interactions between allocation group and maternal pre‐pregnancy BMI, height, age, or fetal sex. At visit 2, there were significant interactions between parity and allocation group for BPD (*p* = .02) and FL (*p* = .04). The intervention effect on BPD and FL became more positive as parity increased (Figure 4). Fetal BPD and FL were smaller among primiparous women and larger among women with more than one previous delivery in the treatment group than in the control group. The supplement had no significant effect on growth of any of the fetal biometry measures considered (Table 2). There were no interactions between allocation group and maternal characteristics or fetal sex.

**Table 2:** Regression coefficients and 95% confidence intervals (CIs) derived from mixed effect regression models analysing the effect of allocation group on fetal growth variables in the intention‐to‐treat and per‐protocol analyses. Models were adjusted for sex, gestational age (GA), and GA^2^

	Intention‐to‐treat analysis		Per‐protocol analysis	
	Estimate (95% CI)	*p*	Estimate (95% CI)	*p*
HC (cm)	−0.06 (−0.15, 0.04)	.27	−0.05 (−0.16, 0.05)	.33
BPD (cm)	−0.01 (−0.04, 0.02)	.61	−0.01 (−0.05, 0.03)	.65
AC (cm)	0.00 (−0.12, 0.11)	.93	−0.01 (−0.13, 0.11)	.88
FL (cm)	0.00 (−0.03, 0.03)	.92	0.00 (−0.03, 0.03)	.88

*Note*. All values are regression coefficients; 95% CIs are reported in parentheses. AC = abdominal circumference; BPD = biparietal diameter; FL = femur length; HC = head circumference.

### 3.2 Per‐protocol analysis

Findings were similar to the ITT analysis. Neither CRL at visit 1 (treatment mean CRL: −0.03SD; control mean CRL: 0.02SD; difference between means: 0.05SD, 95% CI [−0.09SD, 0.18SD]; *p* = .51) nor HC, BPD, FL, and AC at subsequent visits (Figure 3) were significantly different between allocation groups. As in the larger group of women, there was a significant interaction between allocation group and parity (Figure 4) for FL at visit 2 (*p* = .03); however, the interaction was not significant for BPD. The intervention did not have a significant effect on growth of HC, AC, and FL.

### 3.3 Compliance

Throughout pregnancy, the percentage of compliant women was higher in the control group than in the treatment group. In the ITT analysis, 56% of women were compliant at visit 1 (treatment: 51%, control: 61%), and 58% were compliant at visits 2 (treatment: 50%, control: 64%) and 3 (treatment: 50%, control: 66%). The percentage of compliant women decreased in the PP analysis (52%, 54%, and 55% at visits 1, 2, and 3, respectively). Less than 50% of the women in the treatment group (45%, 45%, and 47% at visits 1, 2, and 3, respectively) and approximately 60% of women in the control group were compliant (58%, 61%, and 62% in each of the three visits). Compliance had no significant effect on fetal size and growth at any stage of pregnancy (results not shown). Adding compliance to the regression models described above did not change the significance or the direction of the associations.

## 4 DISCUSSION

In a food‐based randomised controlled trial among Indian women living in Mumbai slums, a daily micronutrient‐rich snack eaten preconceptionally and throughout pregnancy had no effect on ultrasound measures of fetal size or growth. At visit 2 (19–21 weeks), BPD and FL were significantly influenced by an interaction between allocation group and parity, with the supplement having a greater positive effect in fetuses of multiparous women. Fetal measures were positively correlated throughout gestation with the highest correlations observed between visits 2 (19–29 weeks) and 3 (28–32 weeks). At all stages of pregnancy, fetal AC was smaller than the INTERGROWTH‐21^st^ international standard, while HC and FL were comparable.

### 4.1 Strengths and limitations

Strengths of the study were individual random assignment and supervised supplementation. Employing health workers from the community maximised participation. Estimating gestational age using the LMP date instead of by ultrasound enabled the detection of possible variations of fetal size in early pregnancy. The serial monitoring of menstrual period dates as well as the additional inclusion criteria placed on the LMP minimised the possibility of computing erroneous gestational ages. The timing of supplementation (3–6 pm) was chosen to minimise interference with the women's normal diet. Comparison of the women's food intakes before pregnancy and in the second trimester will be the topic of a separate manuscript. There were no differences in baseline or second trimester food intakes and no differences in the changes in food intakes between baseline and second trimester, between allocation groups (results not shown here).

There were several limitations of the study. In total, 34% of women were lost to follow‐up; however, a detailed analysis showed that the differences between women who stayed and those that dropped out were small and did not differ between allocation groups (Potdar et al., 2014). Only 898 (39%) women had completed the data for the ultrasound measures and the baby's sex, and there were significant differences in occupation and parity between those with complete and incomplete scan data. Missing data might have reduced the accuracy of the estimate of the intervention effect. The scheduling of the last scan at 28–32 weeks of gestation meant that we could not fully assess possible effects of the supplement during the last trimester of pregnancy. Repeat measures of CRL, HC, BPD, and FL were not collected; thus, the available data might be subject to measurement error. However, studies have shown a high degree of repeatability of CRL, HC, BPD, and FL (Perni et al., 2004; Souka, Pilalis, Papastefanou, Kassanons, & Kassanons, 2012) and stable variability throughout gestation of z‐scores of these ultrasound measures (Sarris et al., 2012). The most variable measurement (AC) was measured 3 times if the radiologist did not have a perfect view.

### 4.2 Interpretation of the main findings

Fetal ultrasound measures were analysed to understand whether and at what stage of pregnancy the supplement influenced fetal growth. We have reported that the supplement increased birth weight and other “soft tissue” measurement (skinfolds and abdominal, mid‐upper arm and chest circumference) in the newborns of mothers supplemented for ≥3 months before pregnancy but had no effect on “bony measurements” (length and head circumference) (Potdar et al., 2014). Independently of length of supplementation, the effects on birth weight and soft tissue measurements were modified by maternal pre‐pregnancy BMI (there were greater effects of the supplementation among women of normal or high BMI). In contrast, we were unable to detect an effect on ultrasound measures of fetal size and growth, and there was no evidence of an interaction between allocation group and BMI.

These differences between fetal and newborn findings may be partially explained by the nature of the ultrasound measures. HC, BPD, and FL are measures of bone size. AC, the only soft tissue measure available, is characterised by high variability and can be distorted by the transducer, although care was taken to avoid this. To understand whether improving maternal nutrition has a significant effect on fetal growth, additional ultrasound measures of fetal soft tissues, such as mid‐thigh muscle thickness and abdominal subcutaneous tissue (O'Connor et al., 2013), may be informative. Fetal growth during late gestation (>33 weeks) might also explain the differences in supplement effects between fetal and birth measures (Bernstein, Goran, Amini, & Catalano, 1997; Tanner, 1978). The sample in this study was smaller than the one used to analyse the effect of the supplement on birth weight; however, the differences observed between fetal and newborn findings could not be explained by the available sample size as we still found an effect of the supplement on birthweight and an interaction between allocation group and maternal pre‐pregnancy BMI in this study sample (results not shown).

Numerous trials have examined the effect of multiple micronutrient supplementation during pregnancy on birth outcomes (Brough, Rees, Crawford, Morton, & Dorman, 2010; Osrin et al., 2005; Zagré, Desplats, Adou, Mamadoultaibou, & Aguayo, 2007), but literature on the effect of supplementation on fetal biometry is scarce. A randomised controlled trial in The Gambia showed that a prenatal lipid‐based nutritional supplement had no effect on fetal growth overall, but that fetal measurements at 30‐week gestation were larger in the dry season among women receiving protein and energy compared with those receiving multiple micronutrients alone or with protein and energy and multiple micronutrients combined (Johnson et al., 2016). A trial in Peru showed a positive effect of prenatal zinc supplementation on FL (Merialdi et al., 2004); however, the quantity of zinc used (25 mg) was more than 27 times higher than that in our food‐based supplement (0.9 mg). An observational study in the Netherlands linked higher maternal cow's milk consumption in the first trimester of pregnancy with increased fetal weight gain but found no associations between milk consumption and fetal HC and FL (Heppe et al., 2011). To our knowledge, there are no similar studies for GLV and fruit.

### 4.3 Visit 2 biometry: interaction with maternal parity

We found an interaction between intervention group and parity in relation to fetal BPD and FL at visit 2 (19–21 weeks). Among primiparous women, fetal size was smaller in the intervention group than in controls, and the opposite was true among women with more than one previous delivery. Since there were no similar interactions at visit 1 (9–12 weeks) or visit 3 (28–32 weeks), this suggests that fetal growth was slower in the intervention group in early pregnancy among women of lower parity, but “caught up” between visits 2 and 3, or that it was faster in early pregnancy among women of higher parity and became slower between visits 2 and 3. The observed effect should be interpreted with caution as it might be a chance finding. In our study, as expected, birth weight increased with parity, with the greatest increase in means between first and second births (results not shown). A trial in Burkina‐Faso showed that the effect of multiple‐micronutrient supplementation on birth measurements was greater among multiparous women (Roberfroid et al., 2008); however, no literature was found reporting such an effect on fetal biometry.

### 4.4 Secondary analyses

There were sex‐related differences in CRL, HC, BPD, and AC, with males having larger measurements than females (Table S3 and Figure S1). Similar to previous findings in high‐income countries, these differences in HC, BPD, and AC started from early pregnancy and continued throughout gestation, while FL was similar for both sexes throughout pregnancy (Shwarzler, Bland, Holden, Campbell, & Ville, 2004). We found moderate positive correlations between fetal ultrasound measures through pregnancy. Consistent with studies in other populations (Gaillard, Steegers, de Jongste, Hofman, & Jaddoe, 2014), the strongest correlations were found between adjacent visits, while low correlations were observed between CRL at visit 1 (9–12 weeks) or fetal measurements at visit 2 (19–21 weeks) and birth measurements. The comparison between our population's fetal ultrasound measures and the international standards developed by the INTERGROWTH‐21^st^ project showed that MMNP fetuses had a smaller AC but similar HC and FL. This is consistent with previous studies examining differences in fetal growth between Indian and European populations (Kinare et al., 2010).

## 5 CONCLUSIONS

Overall, supplementation with foods rich in micronutrients did not increase fetal size and growth as measured by standard ultrasound techniques, at any stage of pregnancy, in this study. Future studies should include measures of fetal soft tissues and incorporate fetal biometry in the late third trimester.

## CONFLICTS OF INTEREST

The authors declare that they have no conflicts of interest.

## CONTRIBUTIONS

RPD, CHDF, BMM, and AAJ designed the research. CDG and CHDF wrote the manuscript. AL, RDP, SAS, MG, HC, HS, and SHK conducted the research. EM‐Z and CDG analysed the data. All the authors read and approved the final manuscript.

## Supporting information


**Table S1.** Ingredients of the snack at each stage of the trial
**Table S2.** Mean nutrient composition and mean percentage contribution to nutrient requirements of the snacks at each stage of the trial^a^
**Table S3.** Comparison of z‐scores for HC, BPD, AC, and FL between male and female fetuses
**Table S4.** Comparison of baseline characteristics between women who had 3 scans and women with less than 3 scans. Both groups include pregnant women regardless of whether they satisfy the last menstrual period date conditions imposed for the analysis
**Table S5.** Partial correlations between gestation‐adjusted fetal measures estimated controlling for sex and allocation group. 95% confidence intervals are reported in parenthesis
**Figure S1.** Plots of HC, BPD, AC, and FL according to gestational age (weeks) and fetal sex
