# Supplementary material for: Effect of a micronutrient‐rich snack taken preconceptionally and throughout pregnancy on ultrasound measures of fetal growth: The Mumbai Maternal Nutrition Project (MMNP)
Source: Matern Child Nutr. 2017 Mar 2;14(1):e12441. doi: 10.1111/mcn.12441 (PMC5482394; doi:10.1111/mcn.12441)
Supplement: Supplementary file 1 — Table S1. Ingredients of the snack at each stage of the trial Table S2. Mean nutrient composition and mean percentage contribution to nutrient requirements of the snacks at each stage of the triala Table S3. Comparison of z‐scores for HC, BPD, AC, and FL between male and female fetuses Table S4. Comparison of baseline characteristics between women who had 3 scans and women with less than 3 scans. Both groups include pregnant women regardless of whether they satisfy the last menstrual period date conditions imposed for the analysis Table S5. Partial correlations between gestation‐adjusted fetal measures estimated controlling for sex and allocation group. 95% confidence intervals are reported in parenthesis Figure S1. Plots of HC, BPD, AC, and FL according to gestational age (weeks) and fetal sex [file MCN-14-e12441-s001.docx]

**Table 1S:** Ingredients of the snack at each stage of the trial

|  | **Treatment** | | | | | | | | | **Control** | | |
| --- | --- | --- | --- | --- | --- | --- | --- | --- | --- | --- | --- | --- |
|  |  | |  | |  | | **Fruit bar** | |  | |  |  |
|  | **Jan 2006 to Oct 2006** | | **Oct 2006 to Jun 2007** | | **Jun 2007 to May 2012** | | **Jan 2010 to May 2012** | |  | |  | **Jan 2006  to May 2012** |
| **Ingredients** |  |  |  |  |  |  |  |  |  | |  |  |
| Dry GLV powder (g)^a^ | 7.5 |  | 3.8 |  | 0 |  | 0 |  |  | |  | 0 |
| Milk powder (g) | 16 |  | 12 |  | 12 |  | 0 |  |  | |  | 0 |
| Fruit powder (g) | 4 |  | 4 |  | 0 |  | 0 |  |  | |  | 0 |
| Fresh GLV (g) | 0 |  | 29 |  | 30 |  | 0 |  |  | |  | 0 |
| Dried fruit (g) | 0 |  | 0 |  | 4 |  | 60 |  |  | |  | 0 |
| Chickpeas (g) | 0 |  | 0 |  | 0 |  | 2 |  |  | |  | 0 |
| Sesame seeds (g) | 0 |  | 0 |  | 0 |  | 3 |  |  | |  | 0 |
| Low-micronutrient vegetables ^b^ | 0 |  | 0 |  | 0 |  | 0 |  |  | |  | 18 |
| Binding ingredients (g) ^c^ | 30 |  | 28 |  | 30 |  | 0 |  |  | |  | 22 |
| Spices (g) | 2 |  | 2 |  | 2 |  | 2 |  |  | |  | 2 |

^a^GLV: green leafy vegetable; GLVs included spinach, colocasia, amaranth, fenugreek, coriander, shepu, onion stalk and curry leaves. Dried GLVs were air-dried at room temperature and supplied as powders or flakes. ^b^Low micronutrient vegetables included potato and onion. ^c^Binding ingredients used were wheat flour, rice flour, chickpea flour or semolina. The treatment snacks changed during the course of the trial in order to improve the palatability of the snacks, The nutrient content remained similar (Table 2S).

**Table 2S:** Mean nutrient composition and mean percentage contribution to nutrient requirements of the snacks at each stage of the trial ^a^.

|  | Treatment | | | |  | January 2006 to May 2012  (all snacks) | |
| --- | --- | --- | --- | --- | --- | --- | --- |
|  | January 2006 to October 2006 | October 2006 to June 2007 | June 2007 to May 2012 | January 2010 to May 2012  (fruit bar ^b^) |  | Treatment | Control |
| **Micronutrient content/snack** |  |  |  |  |  |  |  |
| β-Carotene (RE) | 114 + 26 ^c^ | 200 + 23 | 141 + 85 | 353 + 180 |  | 159 + 55 (21-595) ^d^ | 2 + 1 (0-3) |
| Riboflavin (mg) | 0.20 + 0.01 | 0.21 + 0.02 | 0.15 + 0.03 | 0.04 + 0.02 |  | 0.16 + 0.04 (0.00-0.22) | 0.01 + 0.01 (0.00-0.02) |
| Folate (µg) ^e^ | 26.0 + 5.7 | 50.8 + 19.5 | 67.5 + 30.6 | 40.2 + 35.9 |  | 58.5 + 14.6 (5.2-93.0) | 6.1 + 4.6 (2.7-12.1) |
| Vitamin C (mg) | < 1 + 0.0 | 0.5 + 0.6 | 2.1 + 3.0 | 8.7 + 12.7 |  | 2.1 + 1.8 (0.0-36.6) | 0.0 + 0.0 (0.0 – 0.60) |
| Vitamin B12 (µg) | 0.64 + 0.05 | 0.58 + 0.16 | 0.31 + 0.13 | 0.14 + 0.15 |  | 0.38 + 0.14 (0.00-0.74) | 0.18 + 0.25 (0.00-0.60) |
| Calcium (mg) | 210 + 14 | 275 + 66 | 194 + 35 | 76 + 16 |  | 200 + 42 (52-356) | 25 + 35 (8-87) |
| Iron (mg) | 6.85 + 1.07 | 5.90 + 1.58 | 3.93 + 1.26 | 1.75 + 0.49 |  | 4.42 + 1.27 (1.22-7.59) | 0.90 + 0.26 (0.16-1.28) |
| **Macronutrient content/snack ^f^** |  |  |  |  |  |  |  |
| Energy (MJ) | 0.74 + 0.09 | 0.70 + 0.06 | 0.61 + 0.07 | 0.92 + 0.04 |  | 0.69 + 0.08 (0.56-0.92) | 0.37 + 0.05 (0.27-0.66) |
| Protein (g) | 7.3 + 0.9 | 6.9 + 0.7 | 6.4 + 1.0 | 2.7 + 0.3 |  | 6.4 + 1.0 (2.7-7.9) | 2.4 + 0.6 (1.0-3.3) |
| **Percentage of RNI ^g^** |  |  |  |  |  |  |  |
| β-Carotene | 14 | 25 | 18 | 44 |  | 20 | <1 |
| Riboflavin | 14 | 15 | 11 | 3 |  | 11 | <1 |
| Folate | 4 | 8 | 11 | 7 |  | 20 | 1 |
| Vitamin C | <1 | 1 | 4 | 16 |  | 4 | <1 |
| Vitamin B12 | 25 | 22 | 12 | 5 |  | 15 | 7 |
| Calcium | 18 | 23 | 16 | 6 |  | 17 | 2 |
| Iron | 35 | 30 | 20 | 9 |  | 25 | 5 |

^a^ RE, retinol equivalents. RNI, reference nutrient intake

^b^ An uncooked fruit bar was introduced as a treatment snack once per week from January 2010.

^c^ Mean + SD (all such values)

^d^ Weighted mean + SD: range in parenthesis. The weighted average was based on the number of days that the snacks were distributed over the study period. The range is the lowest and the highest nutrient contents measured in a sample of an individual snack.

^e^ Total folate

^f^ Macronutrient content calculated from Indian Food Tables (Gopalan, Rama Sastri, & Balasubramanian, 2000).

^g^ WHO/FAO recommended Reference Nutrient Intakes during the first trimester of pregnancy except for calcium for which only a third trimester value was available (Food and Agriculture Organization & World Health Organization, 2004)

**Table 3S:** Comparison of z-scores for HC, BPD, AC, and FL between male and female fetuses.

|  | **Intention-to-treat Analysis** | | | | **Per-protocol Analysis** | | | |
| --- | --- | --- | --- | --- | --- | --- | --- | --- |
|  | **Boys** | **Girls** | **Difference in means**  **(95% CI)** | **p** | **Boys** | **Girls** | **Difference in means**  **(95% CI)** | **P** |
|  | **Mean (SD)** | **Mean (SD)** |  |  | **Mean (SD)** | **Mean (SD)** |  |  |
| **Visit 1: Adjusted CRL z-score** | 0.08  (1.01) | -0.09  (0.99) | 0.17  (0.05, 0.29) | 0.004 | 0.07  (1.00) | -0.07  (1.00) | 0.14  (0.01, 0.28) | 0.03 |
| **Visit 2: HC LMS z-score** | 0.12  (0.99) | -0.26  (0.95) | 0.38  (0.25, 0.52) | <0.001 | 0.16  (0.98) | -0.20  (0.95) | 0.36  (0.22, 0.49) | <0.001 |
| **Visit 2: BPD LMS z-score** | 0.14  (1.00) | -0.20  (0.98) | 0.34  (0.21, 0.48) | <0.001 | 0.15  (0.98) | -0.19  (0.97) | 0.34  (0.21, 0.48) | <0.001 |
| **Visit 2: FL LMS z-score** | -0.06  (1.02) | 0.09  (1.00) | -0.15  (-0.29, -0.01) | 0.04 | -0.02  (0.99) | 0.02  (1.00) | -0.04  (-0.18, 0.10) | 0.56 |
| **Visit 2: AC LMS z-score** | 0.02  (0.91) | -0.13  (0.98) | 0.15  (0.01, 0.28) | 0.03 | 0.10  (0.95) | -0.11  (0.94) | 0.21  (0.07, 0.33) | 0.002 |
| **Visit 3: HC LMS z-score** | 0.20  (0.99) | -0.24  (0.97) | 0.44  (0.20, 0.58) | <0.001 | 0.24  (0.95) | -0.25  (1.04) | 0.49  (0.36, 0.63) | <0.001 |
| **Visit 3: BPD LMS z-score** | 0.18  (0.99) | -0.23  (0.95) | 0.45  (0.28, 0.55) | <0.001 | 0.22  (0.95) | -0.22  (1.03) | 0.44  (0.31, 0.58) | <0.001 |
| **Visit 3: FL LMS z-score** | 0.02  (1.04) | 0.01  (1.01) | 0.01  (-0.12, 0.16) | 0.83 | -0.01  (1.01) | 0.03  (1.02) | -0.04  (-0.18, 0.10) | 0.59 |
| **Visit 3: AC LMS z-score** | 0.09  (1.10) | -0.09  (1.09) | 0.18  (0.03, 0.33) | 0.02 | 0.12 (1.00) | -0.12  (0.99) | 0.24  (0.11, 0.36) | <0.001 |

^1^ Comparisons were made using two sample t-tests. CRL, crown rump length. HC, head circumference. BPD, biparietal diameter. AC, abdominal circumference. FL, femur length.

CRL measures were adjusted based on the median gestational age at visit 1.

**Table 4S:** Comparison of baseline characteristics between women who had 3 scans and women with less than 3 scans. Both groups include pregnant women regardless of whether they satisfy the last menstrual period date conditions imposed for the analysis.

|  |  | **All three scans (n = 1105)** | | **Less than 3 scans (n = 1186)** | | **p** |
| --- | --- | --- | --- | --- | --- | --- |
|  |  | **Median (IQR)**  **or n(%)** | **N** | **Median (IQR)**  **or n(%)** | **N** |  |
| **Weight (kg)** |  | 45.4 (40 – 51.7) | 1105 | 45.9 (40.6 – 52) | 1185 | 0.16 |
| **Height (cm)^a^** |  | 151.3 (5.46) | 1105 | 151.4 (5.50) | 1185 | 0.49 |
| **BMI (kg/m^2^)** |  | 19.9 (18.0 – 22.6) | 1275 | 19.8 (17.8 – 22.5) | 1184 | 0.25 |
| **Age (years)** |  | 24 (21 – 27) | 1105 | 24 (21 – 27) | 1186 | 0.14 |
| **Parity** |  |  |  |  |  | <0.001 |
|  | 0 | 317 (28.7%) |  | 417 (35.1%) |  |  |
|  | 1 | 560 (50.7%) |  | 501 (42.4%) |  |  |
|  | 2+ | 228 (20.6%) |  | 268 (22.6%) |  |  |
| **Religion** |  |  |  |  |  | 0.32 |
|  | Hindu | 783 (70.7%) |  | 829 (70.0%) |  |  |
|  | Muslim | 290 (26.3%) |  | 308 (26.0%) |  |  |
|  | Other | 32 (2.90%) |  | 48 (4.00%) |  |  |
| **Education** |  |  |  |  |  | 0.11 |
|  | Primary | 103 (9.34%) |  | 141 (11.9%) |  |  |
|  | Secondary | 944 (85.6%) |  | 979 (82.6%) |  |  |
|  | Graduate | 56 (5.08%) |  | 66 (5.56%) |  |  |
| **Social Living Index^a^ (SLI) score** |  | 25.1 (6.04) | 1081 | 24.7 (6.07) | 1134 | 0.10 |
| **Mothertongue** |  |  |  |  |  | 0.03 |
| Marathi/ Gujarati | | 616 (55.9%) |  | 603 (50.9%) |  |  |
| Hindi/ Punjabi/ Bengali | | 390 (35.4%) |  | 454 (38.3%) |  |  |
|  | Other | 96 (8.71%) |  | 128 (10.8%) |  |  |
| **Occupation^c^** |  |  |  |  |  | 0.01 |
| Unskilled / Semi-skilled | | 202 (18.3%) |  | 167 (14.1%) |  |  |
| Skilled/ Self-employed | | 39 (3.53%) |  | 28 (2.36%) |  |  |
| Semi-Professional/ Professional | | 23 (2.08%) |  | 22 (1.85%) |  |  |
| Not working | | 841 (76.1%) |  | 969 (81.7%) |  |  |
| **Dietary Intake** |  |  |  |  |  |  |
| Milk & milk products (tea excluded) |  |  |  |  |  | 0.17 |
|  | < 1 time/wk | 534 (48.3%) |  | 595 (50.2%) |  |  |
|  | 1 – 6 times/wk | 424 (38.4%) |  | 413 (34.8%) |  |  |
|  | ≥ 7 times/wk | 147 (13.3%) |  | 178 (15.0%) |  |  |
| GLV |  |  |  |  |  | 0.82 |
|  | < 1 time/wk | 257 (23.3%) |  | 287 (24.2%) |  |  |
|  | 1 – 6 times/wk | 818 (74.0%) |  | 870 (73.4%) |  |  |
|  | ≥ 7 times/wk | 30 (2.70%) |  | 29 (2.54%) |  |  |
| Fruit |  |  |  |  |  |  |
|  | < 1 time/wk | 168 (15.2%) |  | 207 (17.5%) |  | 0.33 |
|  | 1 – 6 times/wk | 763 (69.1%) |  | 792 (66.8%) |  |  |
|  | ≥ 7 times/wk | 174 (15.7%) |  | 187 (15.8%) |  |  |

^a^ Mean (SD) are presented instead of median (IQR) for normally distributed variables.

Differences between groups were determined using t tests and Mann Whitney test for continuous variables, and Chi-square tests for categorical variables. GLV, green leafy vegetables.

**Table 5S:** Partial correlations between gestation-adjusted fetal measures estimated controlling for sex and allocation group. 95% confidence intervals are reported in parenthesis.

|  |  | **Visit 2** | | | |  | **Visit 3** | | | |  | **Birth** | |
| --- | --- | --- | --- | --- | --- | --- | --- | --- | --- | --- | --- | --- | --- |
|  |  | HC | BPD | FL | AC |  | HC | BPD | FL | AC |  | HC | AC |
| **Visit 1** |  |  |  |  |  |  |  |  |  |  |  |  |  |
| CRL |  | 0.72  (0.68, 0.75)** | 0.61  (0.56, 0.65)** | 0.58  (0.53, 0.63)** | 0.59  (0.54, 0.64)** |  | 0.46  (0.40, 0.52)** | 0.34  (0.30, 0.42)** | 0.45  (0.39, 0.51)** | 0.38  (0.32, 0.44)** |  | 0.14  (0.07, 0.20)** | 0.05  (-0.02, 0.12) |
| **Visit 2** |  |  |  |  |  |  |  |  |  |  |  |  |  |
| HC |  | - | 0.79  (0.76, 0.81)** | 0.66  (0.63, 0.70)** | 0.72  (0.69, 0.75)** |  | 0.65  (0.61, 0.69)** | 0.48  (0.43, 0.53)** | 0.51  (0.46, 0.56)** | 0.49  (0.45, 0.54)** |  | 0.25  (0.19, 0.31)** | 0.15  (0.09, 0.21)** |
| BPD |  | - | - | 0.55  (0.51, 0.60)** | 0.57  (0.53, 0.61)** |  | 0.52  (0.47, 0.57)** | 0.56  (0.52, 0.61)** | 0.40  (0.34, 0.45)** | 0.40  (0.34, 0.45)** |  | 0.20  (0.13, 0.27)** | 0.12  (0.06, 0.18)** |
| FL |  | - | - | - | 0.58  (0.54, 0.62)** |  | 0.51  (0.46, 0.56)** | 0.36  (0.30, 0.41)** | 0.57  (0.52, 0.61)** | 0.42  (0.36, 0.47)** |  | 0.15  (0.09, 0.21)** | 0.11  (0.04, 0.17)* |
| AC |  | - | - | - | - |  | 0.50  (0.45, 0.54)** | 0.38  (0.32, 0.43)** | 0.45  (0.40, 0.50)** | 0.52  (0.47, 0.57)** |  | 0.19  (0.12, 0.25)** | 0.20  (0.13, 0.26)** |
| **Visit 3** |  |  |  |  |  |  |  |  |  |  |  |  |  |
| HC |  | - | - | - | - |  | - | 0.71  (0.68, 0.74)** | 0.51  (0.46, 0.55)** | 0.59  (0.55, 0.63)** |  | 0.52  (0.48, 0.57)** | 0.26  (0.20, 0.32)** |
| BPD |  | - | - | - | - |  | - | - | 0.41  (0.36, 0.46)** | 0.44  (0.39, 0.49)** |  | 0.39  (0.33, 0.44)** | 0.23  (0.17, 0.29)** |
| FL |  | - | - | - | - |  | - | - | - | 0.47  (0.42, 0.52)** |  | 0.30  (0.24, 0.36)** | 0.19  (0.13, 0.25)** |
| AC |  | - | - | - | - |  | - | - | - | - |  | 0.36  (0.31, 0.42)** | 0.31  (0.25, 0.37)** |
| **Birth** |  |  |  |  |  |  |  |  |  |  |  |  |  |
| HC |  | - | - | - | - |  | - | - | - | - |  | - | 0.43  (0.37, 0.48)** |
| AC |  | - | - | - | - |  | - | - | - | - |  | - | - |

** p < 0.001. * p < 0.05. CRL, crown rump length. HC, head circumference. BPD, biparietal diameter. FL, femur length. AC, abdominal circumference.

**Figure1S:** Plots of HC, BPD, AC and FL according to gestational age (weeks) and fetal sex.


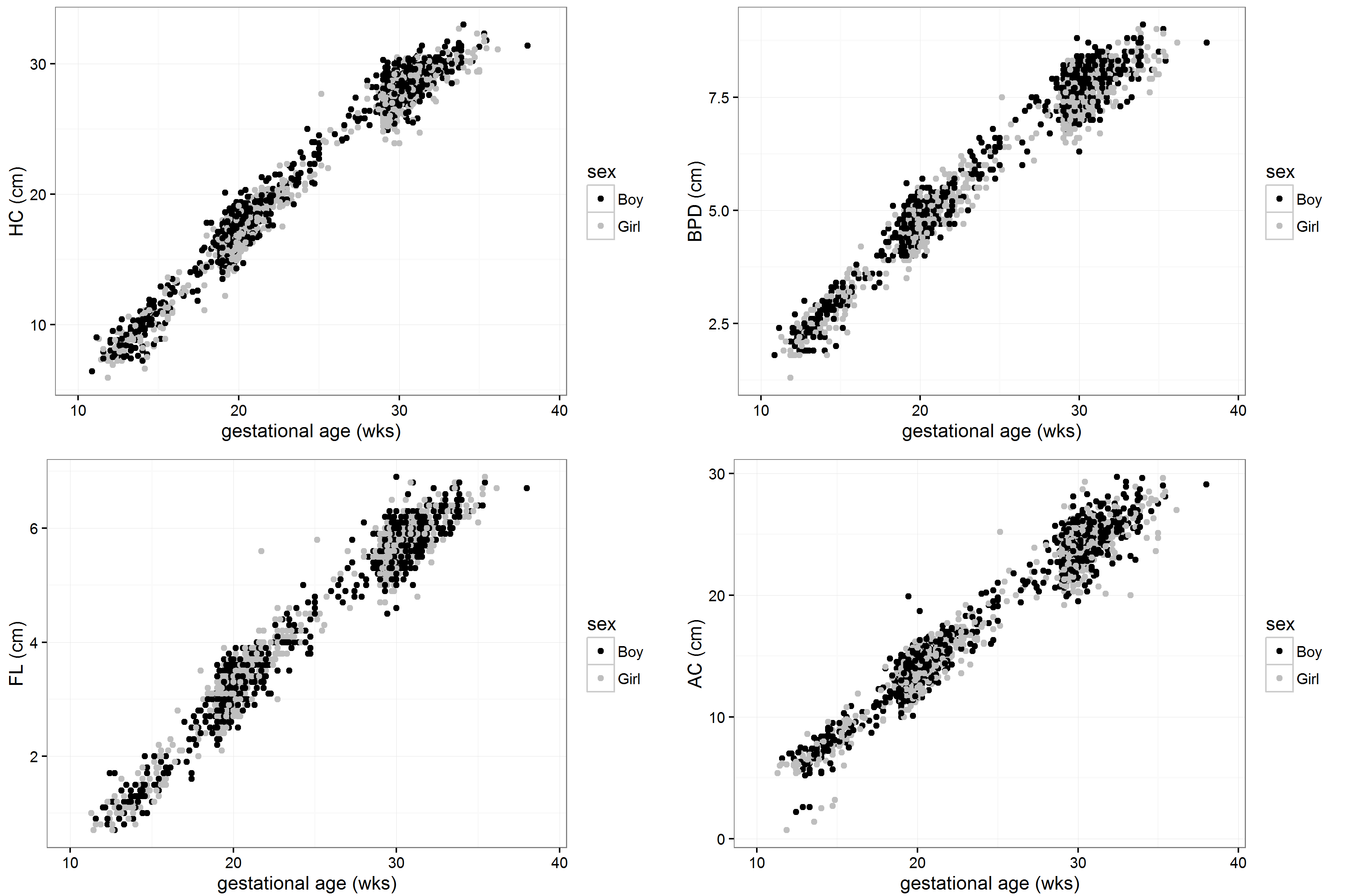


HC, head circumference. BPD, biparietal diameter. FL, femur length. AC, abdominal circumference.

Food and Agriculture Organization, & World Health Organization. (2004). *Joint FAO/WHO expert consulation on human and vitamin requirements* (2nd ed.). Geneva, Switzerland: WHO.

Gopalan, C., Rama Sastri, B., & Balasubramanian, S. (2000). *Nutritive value of Indian Food. Revised and updated by Narasingha Rao BS, Deosthale YG, Pant KC.* Hyderabad, India: National Institute of Nutrition, Indian Council of Medical Research.
